# Experimental Study on the Flow and Heat Transfer Characteristics of TiO_2_-Water Nanofluids in a Spirally Fluted Tube

**DOI:** 10.1186/s11671-017-2284-5

**Published:** 2017-09-02

**Authors:** Cong Qi, Chunyang Li, Guiqing Wang

**Affiliations:** School of Electrical and Power Engineering, China University of Mining and Technology, Xuzhou, 221116 China

**Keywords:** Nanofluids, Spirally fluted tube, Heat transfer enhancement, Frictional resistance coefficient

## Abstract

The flow and heat transfer characteristics of TiO_2_-water nanofluids with different nanoparticle mass fractions in a spirally fluted tube and a smooth tube are experimentally investigated at different Reynolds numbers. The effects of pH values and doses of dispersant agent on the stability of TiO_2_-water nanofluids are discussed. The effects of nanoparticle mass fractions and Reynolds numbers on Nusselt numbers and frictional resistance coefficients in the spirally fluted tube and the smooth tube are also investigated. It is found that TiO_2_-water nanofluids in the spirally fluted tube have a larger enhancement than that in the smooth tube. The heat transfer enhancement and the increase in frictional resistance coefficients of TiO_2_-water nanofluids in the spirally fluted tube and the smooth tube for laminar flow and turbulent flow are compared. It is found that there are a larger increase in heat transfer and a smaller increase in frictional resistance coefficients for turbulent flow than that for laminar flow of TiO_2_-water nanofluids in the spirally fluted tube. The comprehensive evaluations for the thermo-hydraulic performance of TiO_2_-water nanofluids in the smooth tube and spirally fluted tube are also discussed.

## Background

Nanofluids are a type of medium fluids with excellent heat transfer performance (for example ZnO-EG nanofluid [1], Cu-CTAC/NaSal nanofluid [2], MWCNTs-CTAC/NaSal nanofluid [3]), which are applied in various fields, such as clean water generation [4], solar photothermal conversion [5], and boiling heat transfer [6].

Convection heat transfer of nanofluids is an important heat transfer process including natural convection and forced convection heat transfer. Many researchers have investigated the natural convection heat transfer of nanofluids. Li et al. [7] experimentally investigated the natural convection of a square enclosure filled with ZnO-EG/DW nanofluids and obtained a conclusion that the high EG aqueous solution concentration is disadvantageous to heat transfer enhancement. Hu et al. [8] experimentally and numerically investigated the natural convection of Al_2_O_3_-water nanofluids in a square enclosure, and it was found that nanofluids with the highest nanoparticle fraction worsen the heat transfer. He et al. [9] numerically studied the natural convection of Al_2_O_3_-water nanofluids in a square enclosure by a lattice Boltzmann method, and the results showed that the heat transfer performance decreases with the nanoparticle volume fraction. Qi et al. numerically studied the natural convection of Cu-Gallium nanofluids in different aspect ratio enclosures by a single-phase model [10] and a two-phase lattice Boltzmann model [11]; they [12] also studied the natural convection of Al_2_O_3_-water nanofluids using a two-phase lattice Boltzmann model, and the results showed that nanofluids in a smaller aspect ratio enclosure have a higher heat transfer enhancement ratio. In conclusion, it is observed that some factors, such as high heating power and nanoparticle fraction, are advantageous to heat transfer enhancement, while some other factors, such as the big aspect ratio of enclosure and the base fluid with low thermal conductivity, may lead to a reduction in natural convection heat transfer. Although natural convection of nanofluids is widely applied in many fields, it cannot meet the high efficient heat dissipation under the condition of high power density.

Compared with natural convection, forced convection heat transfer has a higher heat transfer coefficient. Researchers adopted different experimental methods to investigate the forced convection heat transfer characteristics of nanofluids. Sun et al. [13, 14] experimentally investigated the flow and heat transfer characteristics of Cu-water, Al-water, Al_2_O_3_-water, Fe_2_O_3_-water, and Cu-water nanofluids in a built-in twisted belt external thread tubes, and it was found that Cu-water nanofluids show the best heat transfer performance. Yang et al. [15] experimentally investigated the flow and heat transfer characteristics of Cu-water and Cu-viscoelastic fluid nanofluids in a smooth tube, and the results showed that Cu-viscoelastic fluid nanofluids have a higher heat transfer performance than viscoelastic base fluid but a lower flow resistance than Cu-water nanofluids. Abdolbaqi et al. [16] experimentally studied the heat transfer enhancement of TiO_2_-BioGlycol/water nanofluids in flat tubes and established a new correlation between the heat transfer enhancement and the friction factor, and the results showed that the heat transfer performance of nanofluids is approximately 28.2% greater than the base fluid. Naphon [17] experimentally studied the heat transfer characteristics of TiO_2_-water nanofluids in horizontal spirally coiled tubes, and it was found that heat transfer performance of nanofluids increases with the decreasing curvature and the increasing nanoparticle fraction. Shahrul et al. [18] and Kumar and Sonawane [19] experimentally investigated the heat transfer characteristics of three kinds of nanofluids (Al_2_O_3_-water, SiO_2_-water, and ZnO-water) and two kinds of nanofluids (Fe_2_O_3_-water and Fe_2_O_3_-EG) in a shell and tube heat exchanger, and it was found that ZnO-water and Fe_2_O_3_-water nanofluids show the best heat transfer performance in their respective research. El-Maghlany et al. [20] experimentally investigated the heat transfer characteristics and pressure drop of Cu-water nanofluids in a horizontal double-tube heat exchanger, and the results showed that heat transfer enhancement of nanofluids increases with the nanoparticle fraction. Sundar et al. [21] experimentally studied the flow and heat transfer characteristics of Fe_3_O_4_-water nanofluids in a horizontal plain tube with return bend and wire coil inserts, and the results showed that heat transfer performance increases with the increasing nanoparticle fraction and decreasing p/d ratio of wire coil inserts. Above studies mainly focused on the heat transfer performance of nanofluids in the smooth tube, flat tube, spirally coiled tube, or tube with wire coil inserts.

In addition to above experimental studies, the forced convection heat transfer characteristics of nanofluids in spirally corrugated tubes are also investigated. Darzi et al. [22, 23] experimentally and numerically studied the turbulent heat transfer of Al_2_O_3_-water nanofluids in a helically corrugated tube, and the results showed that a better heat transfer performance is obtained than that in a plain tube. Darzi et al. [24] experimentally investigated the turbulent heat transfer characteristics of SiO_2_-water nanofluids in helically corrugated tubes and discussed the effects of five pitches of corrugation on the heat transfer of corrugated tubes, and the results showed that the small pitch of corrugations can augment the heat transfer performance significantly. Park et al. [25] studied the heat transfer of thermochromic liquid crystals in a spirally fluted tube, and the results showed that the heat transfer enhancement ratio between the spirally fluted tube and the smooth tube at the low Reynolds number (30,000) is higher than that at high Reynolds numbers (50,000 and 70,000). Above researches mainly investigated the heat transfer and flow characteristics of nanofluids in spirally corrugated tubes. However, the comprehensive analysis for the thermo-hydraulic performance of nanofluids in the smooth tube and spirally fluted tube needs to be discussed further.

Above studies made a great contribution to the flow and heat transfer characteristics in the smooth tube, smooth tube with wire coil inserts, heat exchanger, spirally corrugated tube, and so on. The main novelty of this manuscript mainly includes the following: (1) a new method of testing the stability of nanofluids (transmittance method) is established by an ultraviolet spectrophotometer, which is different from the precipitation method widely adopted by the published references. The results of transmittance method are quantifiable while the results of precipitation method are less quantifiable; and (2) the comprehensive evaluations for the thermo-hydraulic performance of TiO_2_-water nanofluids in the smooth tube and spirally fluted tube are discussed, which are less investigated. On an interesting note, it is found that nanofluids at the highest Reynolds number may not have the best thermo-hydraulic performance in the spirally fluted tube and there is a critical Reynolds number for the best thermo-hydraulic performance.

## Methods

### Nanofluid Preparation and Stability Study

**Fig. 1:**
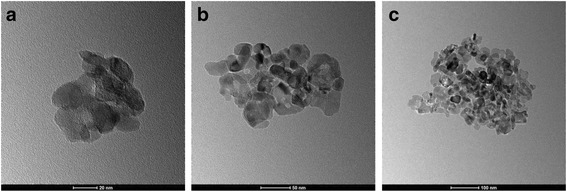
Morphology of TiO_2_ nanoparticles. TEM images of TiO_2_ nanoparticles: **a**  20 nm, **b**  50 nm, and **c**  100 nm

**Fig. 2:**
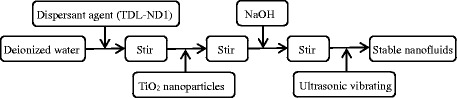
Preparation of nanofluids. Preparation process of TiO_2_-water nanofluids by a two-step method

**Fig. 3:**
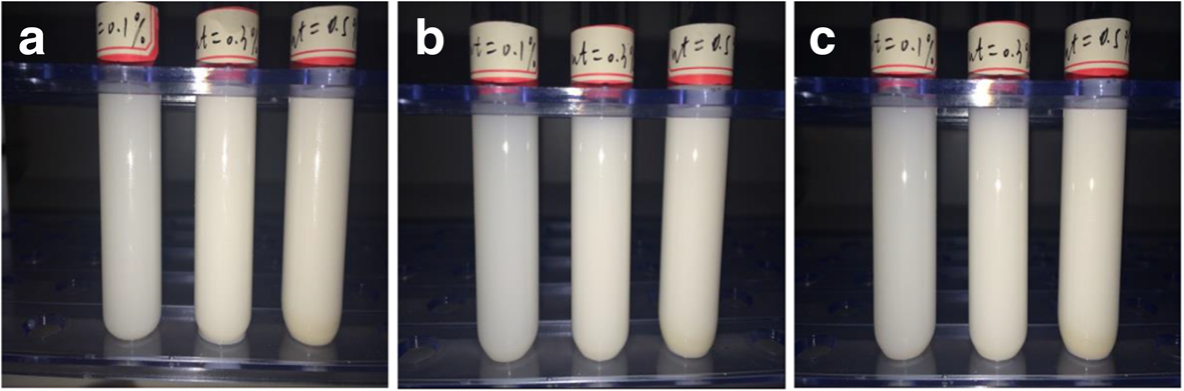
Stability observation of nanofluids. TiO_2_-water nanofluids at different quiescent times: **a** *t* = 0 h, **b** *t* = 48 h, and **c** *t* = 72 h

TiO_2_ is chosen as the nanoparticle, and water is selected as the base fluid. Figure 1 shows the TiO_2_ nanoparticles. TiO_2_-water nanofluids in the experiment are prepared by a two-step method, and Fig. 2 presents the details of the preparation process. For each of the sub-steps, mechanical stirring time is half an hour and the sonication time is 40 min. The mass fraction of the dispersant agent in the water is 6 wt%, and the pH value of nanofluid is 8. Table 1 presents the information of some materials in the preparation process of nanofluids. From Fig. 1, it is found that the nanoparticles easily aggregate together. Hence, the stability of nanofluids is investigated using the precipitation method widely adopted by the published references. The stability of TiO_2_-water nanofluids with various mass fractions (0.1, 0.3, and 0.5 wt%) at different quiescent times is studied in Fig. 3, which shows that the stability of nanofluids 72 h later is still good.

**Table 1:** Information of materials. Information of some materials in the preparation of nanofluids

Materials	Manufacturer	Properties
TiO_2_nanoparticles	Nanjing Tansail Advanced Materials Co., Ltd.	Type: TTP-A10; Crystal form: anatase; Particle diameter: 10 nm
Base fluid (deionized water)	Prepared by ultrapure water device	Resistivity: 16–18.2 MΩ cm@25 °C
Dispersant agent	Nanjing Tansail Advanced Materials Co., Ltd.	Type: TDL-ND1; Element: macromolecule polymers; Scope of application: water or solvent (base fluid)

**Fig. 4:**
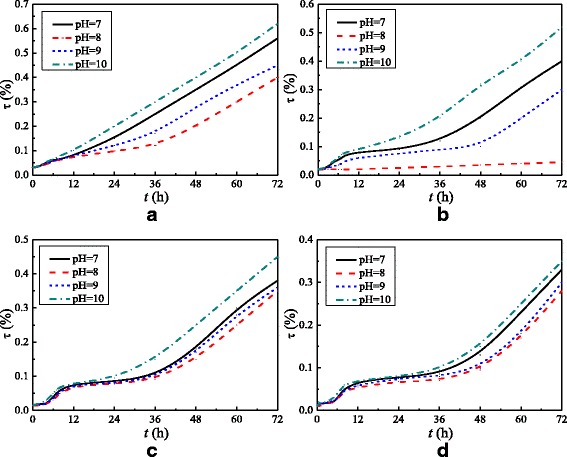
Transmittance (*τ*) of nanofluid (*ω* = 0.3%). Transmittance changes with quiescent time of TiO_2_-water nanofluid (*ω* = 0.3%) with different doses (*M*) of dispersant agent: **a** *M* = 5 wt%, **b** *M* = 6 wt%, **c** *M* = 7 wt%, and **d** *M* = 8 wt%

In order to further check the stability of nanofluids, a new method of testing the stability of nanofluids (transmittance method) is established by an ultraviolet spectrophotometer in this paper. Figure 4 shows the transmittance (*τ*) changes of TiO_2_-water nanofluids (*ω* = 0.3%) with the quiescent time. The effects of different doses (*M*) of dispersant agent and different pH values on the stability of nanofluids are investigated. As we know, if nanoparticles uniformly distribute in the water, nanofluids will reflect the most light, resulting in nanofluids having a high reflectance and a low transmittance. It can be found from Fig. 4 that nanofluids (*ω* = 0.3%) with *M* = 6 wt% and pH = 8 have the lowest transmittance. Nanofluids with other mass fractions (*ω* = 0.1% and *ω* = 0.5%) are all prepared at *M* = 6 wt% and pH = 8 in this paper, and the transmittance change trends of nanofluids with *ω* = 0.1% and *ω* = 0.5% are the same with the nanofluids with *ω* = 0.3%. Hence, the good stability of nanofluids prepared in this paper can be guaranteed. In addition, following the investigation of the effects of dispersant agent and pH on the thermal conductivity and viscosity of water, a small influence on them due to the little dispersant agent and NaOH is found.

**Fig. 5:**
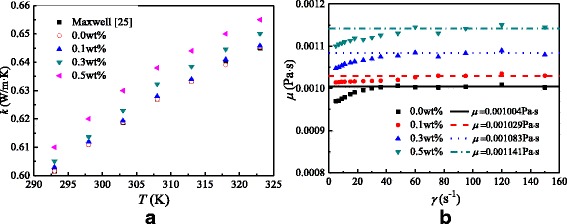
Thermal conductivities and dynamic viscosities. Thermal conductivities and dynamic viscosities of TiO_2_-water nanofluids at different temperatures and shear rates. **a** Thermal conductivities **b** Dynamic viscosity

Figure 5 shows the thermal conductivities and dynamic viscosities of TiO_2_-water nanofluids at different temperatures and shear rates. It is found that the thermal conductivity of water in this paper has a good agreement with Maxwell [26]. It can be found that the thermal conductivity increases with the nanoparticle mass fraction and the thermal conductivity of nanofluids increases by 0.17–1.6% compared with water due to the high thermal conductivity of nanoparticles. Also, it is found that the thermal conductivity increases with the temperature, because high temperature enhances the Brownian motion of nanoparticles and improves the thermal conductivity of nanofluids. In addition to the conclusions of thermal conductivity, it can be found that the dynamic viscosity increases with the shear rate at the initial stage and keeps constant with the increasing shear rate and the viscosity of nanofluids increases by 2.5–13.6% compared with water. It is because that a small shear force added in the nanofluids at the initial stage breaks the equilibrium of the flow field and causes an increase in dynamic viscosity (shear-thickening behavior). The dynamic viscosity is constant when the flow field reaches a steady state again, which has a good agreement with the characteristics of Newtonian fluid.

### Experimental System

**Fig. 6:**
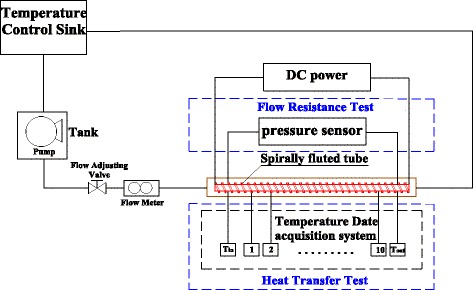
Experimental system. Schematic diagram of experimental system

An experimental system for the flow and heat transfer characteristics of TiO_2_-water nanofluids in a spirally fluted tube is established. Figure 6 represents the schematic diagram of the experimental system. The experimental system mainly includes the heat transfer test section, flow resistance test section, temperature control sink, and pump. The spirally fluted tube is heated by a resistance wire connected to a DC power. Outer wall temperature of the spirally fluted tube is obtained by ten T-type thermocouples which are uniformly distributed in the surface of the spirally fluted tube. Outlet temperature and inlet temperature of nanofluids of the spirally fluted tube are measured by two K-type thermocouples. All thermocouples are connected to a data acquisition instrument (Agilent 34972A). The flow resistance is measured by a differential pressure instrument.

**Fig. 7:**
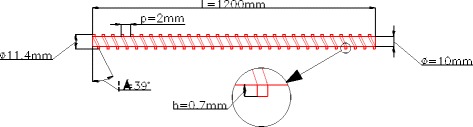
Spirally fluted tube. Detailed diagram of the spirally fluted tube

The detailed diagram of the spirally fluted tube is shown in Fig. 7. For the smooth tube and spirally fluted tube, the materials are all stainless steel, the equivalent diameters are the same, the lengths are all 1200 mm, the test sections are all the middle section 1000 mm of the tube, and 100 mm section is left at each end of the tube in order to avoid the entrance effect.

### Calculation Equations

The heating power is supplied by a DC power:

1 $$ {Q}_{\begin{array}{l}0\\ {}\end{array}}= UI $$

where $$ {Q}_{\begin{array}{l}0\\ {}\end{array}} $$ is the heating power, *U* is the voltage, and *I* is the electric current.

The heat absorbed by fluid is calculated as follows:

2 $$ {Q}_{\mathrm{f}}={c}_{\mathrm{p}}{q}_{\mathrm{m}}\left({T}_{\mathrm{out}}-{T}_{\mathrm{in}}\right) $$

where *Q*_f_ is the heat absorbed by fluid, *c*_p_ is the specific heat of fluid, *q*_m_ is the mass flow rate, and *T*_out_ and *T*_in_ are the outlet temperature and inlet temperature of fluid.

Heat capacity is given as follows:

3 $$ {c}_{\mathrm{p}}=\frac{\left(1-\varphi \right){\left(\rho {c}_{\mathrm{p}}\right)}_{\mathrm{bf}}+\varphi {\left(\rho {c}_{\mathrm{p}}\right)}_{\mathrm{p}}}{\left(1-\varphi \right){\rho}_{\mathrm{bf}}+{\varphi \rho}_{\mathrm{p}}} $$

where *c*_p_ is the heat capacity of nanofluids, *φ* is the nanoparticle volume fraction, the subscript “bf” represents the base fluid, and the subscript “p” represents the nanoparticles.

The average temperature of fluid is calculated as follows:

4 $$ T\mathrm{f}=\left(T\mathrm{out}+T\mathrm{in}\right)/2 $$

where *T*_f_ is the average temperature of fluid in the tube.

The outer wall average temperature of the tube is shown as follows:

5 $$ {T}_{\mathrm{ow}}=\left[\sum_{i=1}^{10}T\mathrm{w}(i)\right]/10 $$

where *T*_ow_ is the outer wall average temperature of the tube, *T*w(*i*) is the temperature of thermocouples attached to the outer wall of tube, and there are ten thermocouples attached uniformly to the outer wall of tube.

The internal wall average temperature of the tube can be calculated as follows:

6 $$ {T}_{\mathrm{iw}}={T}_{\mathrm{ow}}-\frac{Q_{\mathrm{f}}\ln \left(r\mathrm{o}/ ri\right)}{2\pi \lambda l},\left(i=1,2,3\dots 10\right) $$

where *T*_iw_ is the internal wall average temperature of the tube, *r*o and *ri* are the external radius and internal radius of tube, *λ* is the thermal conductivity of tube, and *l* is the length of tube.

The convective heat transfer coefficient is calculated as follows:

7 $$ {h}_{\mathrm{f}}=\frac{Q_{\mathrm{f}}}{\pi {d}_{\mathrm{e}}l\left({T}_{\mathrm{iw}}-{T}_{\mathrm{f}}\right)} $$

where *h*_f_ is the convective heat transfer coefficient and *d*_e_ is the equivalent diameter of the tube.

The Nusselt number is calculated as follows:

8 $$ Nu=\frac{h_{\mathrm{f}}{d}_e}{\lambda_{\mathrm{f}}} $$

where *Nu* is the Nusselt number and *λ*_f_ is the thermal conductivity of fluid in the tube measured by a thermal conductivity measuring instrument.

The Reynolds number is shown as follows:

9 $$ \mathit{\operatorname{Re}}=\rho {ud}_e/{\mu}_{\mathrm{f}} $$

where Re is the Reynolds number, *ρ* is the density of fluid, *u* is the velocity of fluid, and *μ*_f_ is the dynamic viscosity of fluid measured by a super rotational rheometer.

Density of nanofluids is shown as follows:

10 $$ \rho =\left(1-\varphi \right){\rho}_{\mathrm{bf}}+{\varphi \rho}_{\mathrm{p}} $$

where *ρ* is the density of the nanofluids, *φ* is the volume fraction of the nanoparticles, *ρ*_bf_ is the density of water, and *ρ*_p_ is the density of the nanoparticles.

Frictional resistance coefficient of fluid is presented as follows:

11 $$ f=\frac{2d\mathrm{e}}{\rho {u}^2}\cdot \frac{\varDelta p}{\varDelta l} $$

where *f* is the frictional resistance coefficient and $\frac{\varDelta p}{\varDelta l}$ is the pressure drop per unit length.

The equation of the comprehensive evaluation between heat transfer and flow resistance is shown as follows [27]:

12 $$ \varsigma =\left(\frac{Nu}{Nu_{\left(\mathrm{bf}+\mathrm{smooth}\  \mathrm{tube}\right)}}\right)/{\left(\frac{f}{f_{\left(\mathrm{bf}+\mathrm{smooth}\  \mathrm{tube}\right)}}\right)}^{\frac{1}{3}} $$

where *ς* is the comprehensive evaluation index.

### Uncertainty Analysis

Experimental errors are caused by the accuracies of the equipment in the experimental system. The corresponding error equations are shown as follows:

13 $$ \frac{\delta Nu}{Nu}=\sqrt{{\left(\frac{\delta {Q}_{\boldsymbol{f}}}{Q_{\boldsymbol{f}}}\right)}^2+{\left(\frac{\delta T}{T}\right)}^2} $$

14 $$ \frac{\delta f}{f}=\sqrt{{\left(\frac{\delta p}{p}\right)}^2+{\left(\frac{\delta l}{l}\right)}^2+{\left(\frac{\delta q\mathrm{m}}{q\mathrm{m}}\right)}^2} $$

where the accuracy of DC power is ± 5.0%, the accuracy of thermocouple is ± 0.1%, and the error of the Nusselt number can be obtained from Eq. (13) and is approximately ± 5.0%. The accuracy of pressure transducer is ± 0.5%, the accuracy of length is ± 0.1%, the accuracy of mass flow rate is ± 1.06%, and the error of frictional resistance coefficient can be obtained from Eq. (14) and is approximately ± 1.29%.

## Results and Discussions

### Experimental System Validation

**Fig. 8:**
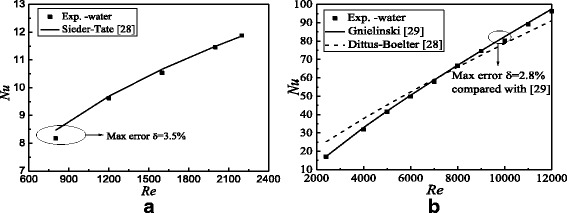
Heat transfer characteristics validation. Comparison of Nusselt numbers between the experimental results and the results of literatures. **a** Laminar flow **b** Turbulent flow

**Fig. 9:**
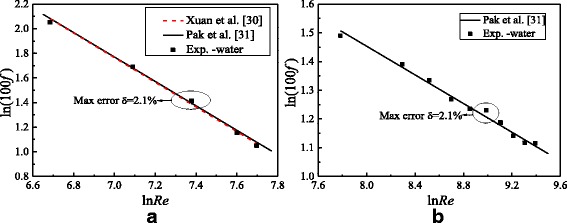
Flow characteristics validation. Comparison of frictional resistance coefficients between the experimental results and the results of literatures. **a** Laminar flow **b** Turbulent flow

Before the experimental study on nanofluids, the experimental system validation is necessary. Water is chosen as the heat transfer medium. Nusselt numbers and frictional resistance coefficients between the experimental results of this paper and the results of published literatures are shown in Figs. 8 and 9. It can be found from Figs. 8 and 9 that Nusselt numbers and frictional resistance coefficients at different Reynolds numbers have a good agreement with the results of the published literatures [28, 29] and [30, 31] respectively. The max errors for Nusselt numbers and frictional resistance coefficients at laminar flow and turbulent flow are approximately 3.5, 2.8, 2.1, and 2.1%, respectively, which verify the accuracy and reliability of the experimental system. Also, it is found that the results of Dittus-Boelter in Fig. 8b are higher than the real results under the transitional flow because the empirical formula only can be applied to the strong turbulence zone, which agrees with the results of literature [28]. It proves the validity of the results in this paper further.

### Experimental Results and Discussions

**Fig. 10:**
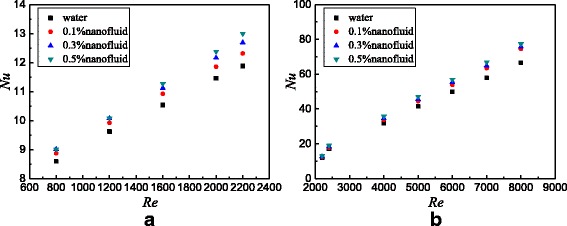
Nusselt numbers in the smooth tube. Nusselt numbers of the smooth tube filled with nanofluids at different Reynolds numbers. **a** Laminar flow **b** Turbulent flow

The flow and heat transfer characteristics of TiO_2_-water nanofluids in the smooth tube are investigated. Figure 10 presents the Nusselt numbers of the smooth tube filled with nanofluids at different Reynolds numbers. For laminar flow and turbulent flow, the Nusselt number increases with the Reynolds number and nanoparticle mass fraction. The turbulivity of fluid increases with the Reynolds number, which reduces the laminar boundary layer and improves the heat transfer. Adding more nanoparticles into the base fluid causes the increase of whole thermal conductivity, which also improves the heat transfer. In addition, it is suggested [32, 33] that other factors including the increase of Brownian motion of nanoparticles, reduction of the contact angles, non-uniform shear rate, particle shape, and aggregation also have great influence in the heat transfer enhancement. In the previous published paper [11], the effects of Brownian force and particle size on heat transfer enhancement were discussed. It was found that Brownian force is the biggest force of the interaction forces between nanoparticles, which is advantageous to the heat transfer enhancement, and the small particle size is also advantageous to the heat transfer enhancement. It is found from Fig. 10a that the heat transfer enhancement ratio from water to *ω* = 0.1 wt% nanofluids shows the largest one, but the heat transfer enhancement ratio of nanofluids from *ω* = 0.1 wt% to *ω* = 0.3 wt% begins to decline, and the heat transfer enhancement ratio of nanofluids from *ω* = 0.3 wt% to *ω* = 0.5 wt% witnesses the smallest one. As Fig. 5 shows, the thermal conductivity and viscosity of nanofluids increase by 0.17–1.6% and 2.5–13.6% compared with water, respectively. For the laminar flow, the effects of viscosity on heat transfer are small due to the low velocity and few nanoparticles, and then the thermal conductivity plays a major role from water to *ω* = 0.1 wt% nanofluids. However, with an increase in nanoparticle fraction, it shows a more dramatic increase in viscosity compared with the increase in the thermal conductivity, which causes the heat transfer enhancement ratio to decrease. For the turbulent flow, it is found that the heat transfer enhancements of nanofluids with different nanoparticle mass fractions are close. This is because the turbulence plays a major role in the heat transfer enhancement, and the effect of nanoparticle mass fraction becomes small. Also, it can be found that nanofluids show a larger heat transfer enhancement ratio in laminar flow compared with that in turbulent flow. Nanoparticle mass fraction plays a major role in the heat transfer enhancement in laminar flow, and it shows a large heat transfer enhancement with the increasing nanoparticle mass fraction. However, the effect of nanoparticle mass fraction on heat transfer enhancement becomes small in turbulent flow, and the turbulence intensity plays a major role; hence, it shows a smaller heat transfer enhancement ratio with the increasing nanoparticle mass fraction in turbulent flow compared with that in laminar flow.

**Fig. 11:**
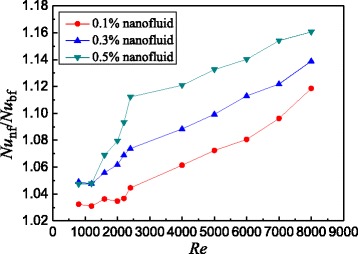
Nusselt number ratios in the smooth tube. Nusselt number ratios between nanofluids and base fluid in the smooth tube

Based on the data of Fig. 10, Fig. 11 shows the Nusselt number ratios of nanofluids to the water in the smooth tube. It can be found that TiO_2_-water nanofluids with *ω* = 0.5 wt%, *ω* = 0.3 wt%, and *ω* = 0.1 wt% enhance the heat transfer by 11.2, 7.4, and 4.5% for laminar flow and 16.1, 13.9, and 11.9% for turbulent flow at best compared with water in the smooth tube, respectively.

**Fig. 12:**
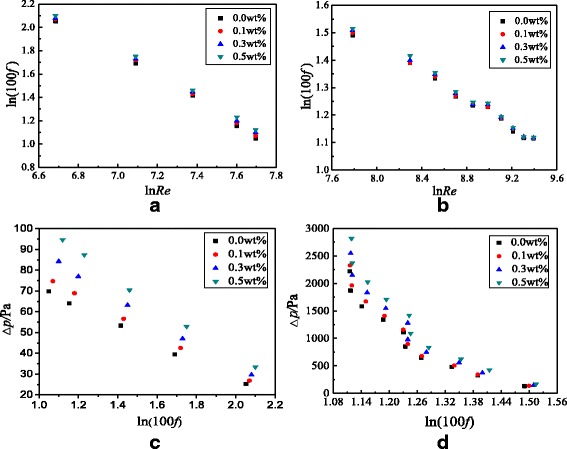
Frictional resistance coefficients and pressure drop in the smooth tube. Frictional resistance coefficients and pressure drop of the smooth tube filled with nanofluids. Frictional resistance coefficients: **a** laminar flow and **b** turbulent flow; pressure drop: **c** laminar flow and **d** turbulent flow

In addition to the study on the heat transfer characteristics of TiO_2_-water nanofluids in the smooth tube, the flow characteristics are also investigated. Figure 12 presents the frictional resistance coefficients and pressure drop of the smooth tube filled with nanofluids. From Fig. 12, it is found that the frictional resistance coefficient decreases with the Reynolds number because the increasing Reynolds number causes the increase of velocity, which is inversely proportional to the frictional resistance coefficient according to the Eqs. (9) and (11). It is found that the pressure drop decreases with the frictional resistance coefficient because the pressure drop is proportional to the Reynolds number but the frictional resistance coefficient is inversely proportional to the Reynolds number. Hence, the pressure drop is inversely proportional to the frictional resistance coefficient. Also, it can be found from Fig. 12 that the frictional resistance coefficient increases with nanoparticle mass fraction but the increase is small between different nanoparticle mass fractions. For TiO_2_-water nanofluids with *ω* = 0.5 wt%, *ω* = 0.3 wt%, and *ω* = 0.1 wt% in the smooth tube, a maximum enhancement of 7.9, 5.2, and 3.0% at laminar flow and 2.5, 1.5, and 0.6% at turbulent flow occurs in the frictional resistance coefficients compared with water in the smooth tube, respectively. Adding nanoparticles into water causes the increase of viscosity which is proportional to the frictional resistance coefficient. However, the frictional resistance is mainly caused by the screw structure of the spirally fluted tube, and the effect of nanoparticles on the frictional resistance is much smaller than that of the screw structure, which causes a small difference between different nanoparticle mass fractions.

**Fig. 13:**
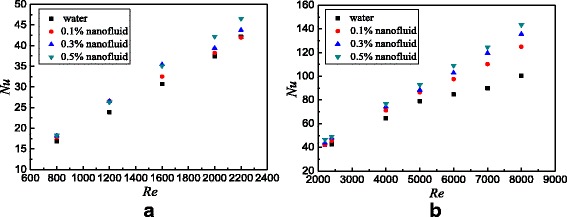
Nusselt numbers in the spirally fluted tube. Nusselt numbers of the spirally fluted tube filled with nanofluids at different Reynolds numbers. **a** Laminar flow **b** Turbulent flow

Above studies are on smooth tube, and the flow and heat transfer characteristics of water and TiO_2_-water nanofluids in the spirally fluted tube will be investigated in the following text. Figure 13 presents the Nusselt numbers of the spirally fluted tube filled with TiO_2_-water nanofluids at different Reynolds numbers. It obtains a similar conclusion in the spirally fluted tube (Fig. 13) similar to that in the smooth tube (Fig. 10). It is found that the Nusselt number increases with the Reynolds number and nanoparticle mass fraction. The differences between the spirally fluted tube and smooth tube are that there is a larger heat transfer enhancement in the spirally fluted tube than that in the smooth tube, which is due to the screw structure of the spirally fluted tube.

**Fig. 14:**
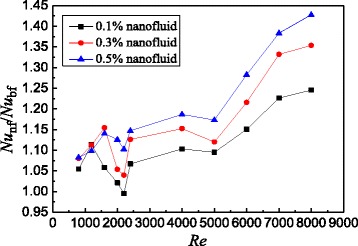
Nusselt number ratios in the spirally fluted tube. Nusselt number ratios between nanofluids and base fluid in the spirally fluted tube

Based on the data of Fig. 13, Fig. 14 shows the Nusselt number ratios of nanofluids to the water in the spirally fluted tube. Figure 14 shows that TiO_2_-water nanofluids with *ω* = 0.5 wt%, *ω* = 0.3 wt%, and *ω* = 0.1 wt% can enhance the heat transfer by 14.7, 12.6, and 11.3% for laminar flow and 42.8, 35.4, and 24.6% for turbulent flow at best compared with water in the spirally fluted tube, respectively. There is a larger increase in heat transfer for turbulent flow than that for laminar flow.

**Fig. 15:**
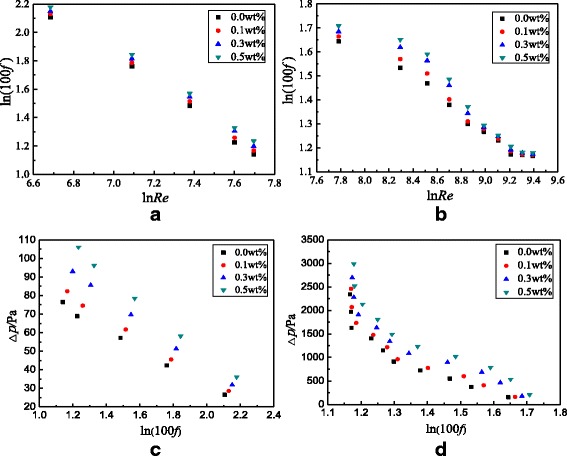
Frictional resistance coefficients and pressure drop in the spirally fluted tube. Frictional resistance coefficients of the spirally fluted tube filled with nanofluids. Frictional resistance coefficients: **a** laminar flow and **b** turbulent flow; pressure drop: **c** laminar flow and **d** turbulent flow

The flow characteristics of TiO_2_-water nanofluids in the spirally fluted tube are also studied. Figure 15 presents the frictional resistance coefficients and pressure drop of the spirally fluted tube filled with nanofluids, which shows that the frictional resistance coefficient decreases with the Reynolds number and increases with the nanoparticle mass fraction, and the pressure drop decreases with the frictional resistance coefficient. The reasons are similar to that in the smooth tube (Fig. 12c, d). TiO_2_-water nanofluids with *ω* = 0.5 wt%, *ω* = 0.3 wt%, and *ω* = 0.1 wt% can enhance the frictional resistance coefficients by 20.2, 16.5, and 12.5% for laminar flow and 10.5, 7.7, and 2.0% for turbulent flow at best compared with water in the spirally fluted tube, respectively. There is a smaller increase in frictional resistance coefficients for turbulent flow than that for laminar flow.

**Fig. 16:**
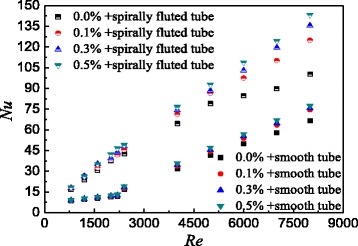
Comparison of Nusselt numbers in two tubes. Comparison of Nusselt numbers between the smooth tube and spirally fluted tube filled with nanofluids at different Reynolds numbers

The heat transfer characteristics of TiO_2_-water nanofluids in the smooth tube and spirally fluted tube are investigated in this paper separately. Figure 16 shows the comparison of Nusselt numbers between the smooth tube and the spirally fluted tube filled with nanofluids at different Reynolds numbers. It can be found that TiO_2_-water nanofluids with *ω* = 0.5 wt%, *ω* = 0.3 wt%, and *ω* = 0.1 wt% in the spirally fluted tube can enhance the heat transfer by 257.9, 245.1, and 240.7% at best compared with TiO_2_-water nanofluids in the smooth tube, respectively. Also, TiO_2_-water nanofluids with *ω* = 0.5 wt%, *ω* = 0.3 wt%, and *ω* = 0.1 wt% in the spirally fluted tube can enhance the heat transfer by 291.3, 268.8, and 253.1% at best compared with water in the smooth tube, respectively. TiO_2_-water nanofluids in the spirally fluted tube have a larger enhancement than that in the smooth tube.

**Fig. 17:**
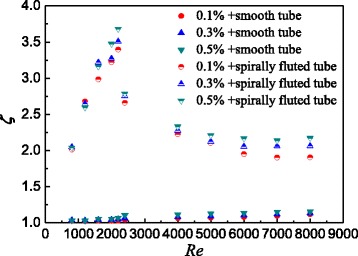
Comprehensive analysis of the two tubes. Comprehensive analysis of nanofluids in the smooth tube and the spirally fluted tube

In order to synthetically analyze the thermo-hydraulic performance of TiO_2_-water nanofluids in the smooth tube and spirally fluted tube, Fig. 17 presents the comprehensive analysis of nanofluids in the smooth tube and the spirally fluted tube based on the Eq. (12). It can be found that the highest comprehensive evaluation index ξ for spirally fluted tube is about at *Re* = 2300 which is the critical Reynolds number between laminar flow and turbulent flow. The increases of the Nusselt number and frictional resistance coefficients are mainly due to the nanoparticles, the Reynolds number, and the screw structure of spirally fluted tube. For spirally fluted tube, due to the screw structure, the increase of the Nusselt number is larger than the increase of frictional resistance coefficients at small Reynolds number (*Re* ≤ 2300); conversely, the increase of the Nusselt number is smaller than the increase of frictional resistance coefficients at big Reynolds number (*Re* > 2300). Also, the comprehensive evaluation index ξ for the smooth tube increases with the Reynolds number. The increase of the Nusselt number is always larger than the increase of frictional resistance coefficients because the smooth tube has no screw structure. The conclusions of Fig. 17 are very important for the choices of tubes and Reynolds numbers in the heat-exchange equipment considering the comprehensive evaluation of the thermo-hydraulic performance. For the smooth tube, the higher Reynolds number can be chosen due to the factor that the thermo-hydraulic index always increases with the Reynolds number. While for the spirally fluted tube, the appropriate Reynolds number for the highest thermo-hydraulic index is about 2300.

## Conclusions

The flow and heat transfer characteristics of TiO_2_-water nanofluids in a spirally fluted tube are experimentally studied. Some conclusions are obtained as follows:

1.  TiO_2_-water nanofluids with different nanoparticle mass fractions are prepared, and TiO_2_-water nanofluids with M = 6 wt% and pH = 8 have the lowest transmittance and show the best stability.
2.  For TiO_2_-water nanofluids in the spirally fluted tube, there is a larger increase in heat transfer and a smaller increase in frictional resistance coefficients for turbulent flow than that for laminar flow.
3.  TiO_2_-water nanofluids in the spirally fluted tube have a larger enhancement than that in the smooth tube. TiO_2_-water nanofluids in the spirally fluted tube can enhance the heat transfer by 257.9% at best compared with that in the smooth tube.
4.  The highest comprehensive evaluation indexes of TiO_2_-water nanofluids in the spirally fluted tube and smooth tube are different. For the spirally fluted tube, the highest comprehensive evaluation index ξ is at *Re* = 2300 which is the critical Reynolds number between the laminar flow and the turbulent flow. For the smooth tube, the comprehensive evaluation index ξ increases with the Reynolds number.
